# Safety and Feasibility of Ultrasound-Guided Access for Coronary Interventions through Distal Left Radial Route

**DOI:** 10.1155/2022/2141524

**Published:** 2022-03-25

**Authors:** Tapan Ghose, Ranjan Kachru, Jaideep Dey, Wasi Ullah Khan, Ratna Sud, Suraiya Jabeen, Shahnawaz Husain, Aparna Pant

**Affiliations:** ^1^Cardiology, Fortis Flt. Lt. Rajan Dhall Hospital, Sector B, Pocket 1, Aruna Asaf Ali Marg, Vasant Kunj, New Delhi-70, India; ^2^Non-Invasive Cardiology, BLK MAX Superspeciality Hospital, Pusa Road, Rajendra Place, New Delhi-05, India

## Abstract

**Aims:**

Left distal transradial arterial approach (ldTRA) is a new interventional route that spares right radial artery (RRA) for use in haemodialysis and as bypass graft. Vasant Kunj Left dIstal Transradial ArtEry approach (VKLITE) study aimed to assess the feasibility and safety of ldTRA access during coronary angiography (CAG) and percutaneous coronary intervention (PCI).

**Methods and Results:**

Between April 2018 and June 2020, 108 patients were enrolled and underwent CAG ± PCI via ultrasound guided ldTRA. Arterial puncture, CAG, and PCI were successful in 96.3% (104/108), 92.1% (93/101), and 94.1% (32/34) patients, respectively. Access site crossover rate was 14/108 (13.0%). Mean puncture, procedure, and haemostasis time (minutes) were 6.7 ± 7.1, 55.7 ± 32.8, and 23.1 ± 11.9. Median total fluoroscopic time was 6.6 minutes (IQR-14.2), and median total radiation dose was 39.2 Gy-cm^2^ (IQR-97.0). Local haematoma occurred in 11 patients (10.2%) with major haematoma in 1.9%, all recovering within three weeks. Mean pain score was 2.4 ± 2.3, and patient satisfaction score was 9.0 ± 1.3. LdTRA access compared with RRA access (*n* = 121) showed significantly increased mean procedure time (55.7 ± 32.8 vs. 43.9 ± 26.0 minutes, *p* = 0.01) and median total fluoroscopic time (6.6 [IQR-14.2] vs. 4.7 [IQR-8.2] minutes, *p* = 0.02), with similar median total radiation dose (39.2 [IQR-97.0] vs. 43.8 [IQR-54.5] Gy-cm^2^, *p* = 0.56). No radial artery loss, dissection, pseudoaneurysm, arteriovenous fistula, or nerve injury was noted.

**Conclusions:**

LdTRA access is feasible with few complications during CAG/PCI. Patient comfort and satisfaction makes it a desirable route for coronary interventions.

## 1. Introduction

Transradial approach (TRA) for coronary intervention has increased rapidly over the last decade. Shorter recovery times with improved morbidity and mortality indicators have led to TRA being endorsed as the preferred access site for myocardial revascularization in current guidelines [1, 2]. Left distal transradial artery approach (ldTRA) is a new route for coronary intervention, first described by Dr. Ferdinand Kiemeneij in 2017 [3]. This spares immobilization and discomfort of right hand, seen after TRA. The course of coronary catheters via left radial artery (LRA) into the aortic root is like femoral arterial access, which the operators are most familiar with. The right radial artery (RRA) is also spared for future use in haemodialysis and as a conduit for coronary artery bypass graft (CABG) surgery.

However, the procedural implications, learning curve, and feasibility with current generation coronary hardware have been infrequently reported in literature [4, 5]. We present a study assessing feasibility and safety of ldTRA route for coronary intervention. To our knowledge, this is the first study from India using routine ultrasound guided left distal radial artery (ldRA) puncture and performing procedural comparison between ldTRA and RRA routes.

## 2. Material and Methods

### 2.1. Study Population and Operators

Vasant Kunj Left dIstal Transradial artEry approach (VKLITE) study was an open level, prospective, observational cohort study. The study was approved by the Institutional Review Board/Independent Ethics Committee of our institution and registered with Clinical Trials Registry of India (registration number: CTRI/2019/07/020002). Target number of patients for enrolment was 200, based on previous studies to ascertain procedural success and associated complications [3, 4]. Inclusion criteria for the study were as follows:

- Patients ≥20 years of age, requiring coronary angiography (CAG)/percutaneous coronary intervention (PCI)
- Having a palpable ldRA
- Providing willful consent for inclusion in study

Exclusion criteria for the study were as follows:

- Subjects with nonpalpable LRA
- Abnormal modified Allen's test
- Pregnant patients

In modified Allen's test, with the hand elevated, patient clenches his/her fist for 30 seconds. Sufficient pressure is applied to both radial and ulnar arteries to occlude blood flow, following which the hand is opened and should appear blanched. While maintaining pressure over radial artery, ulnar pressure is released. If normal hand colour returns within 5–15 seconds, the test is normal, suggestive of adequate ulnar blood flow; otherwise, the test is abnormal, suggestive of compromised ulnar blood flow [6].

All patients provided written informed consent before study participation. Two independent, expert radial operators with 25-year interventional experience performed the procedures.

### 2.2. Procedure

**Figure 1:**
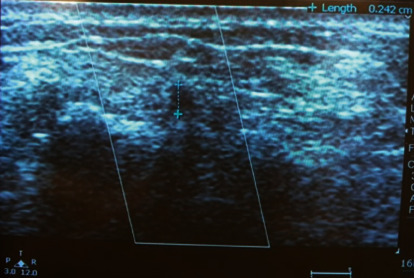
Ultrasound guided left distal radial arterial diameter measurement.

Prior to part preparation, diameter, and flow in LRA was assessed at the wrist, anatomical snuff box (triangular depression on radial aspect of hand bordered by tendon of extensor pollicis longus medially and tendons of extensor pollicis brevis and abductor pollicis longus laterally), and distal vessel using vascular ultrasound (Philips M2540 A Ultrasound system, Bothell, WA, USA). The largest diameter across vascular lumen (inner edge to inner edge) was measured (Figure 1).

**Figure 2:**
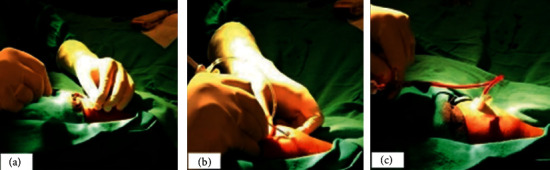
(a): Guidewire inserted via Seldinger technique, (b) arterial sheath inserted over the wire, and (c) arterial backflow in sheath noted.

Under aseptic conditions, the puncture site was infiltrated subcutaneously with 2.0 cc lidocaine (1%) solution. Using the Seldinger technique, radial artery was punctured distal to or at anatomical snuff box using a 20-gauge needle (Spectrum MedTech, Gujarat, India), under ultrasonographic guidance. Dorsal arterial wall was punctured while maintaining the puncture needle at an angle of 30-degree to the skin. On successful puncture, a 0.025ʺ guidewire (Figure 2) was subsequently introduced through the needle, which was exchanged for a 5F radial sheath (Terumo Radiofocus Introducer II, Tokyo, Japan). If PCI was undertaken subsequently, the 5F sheath was exchanged for a 6F sheath. After confirming arterial pressure tracing, 5000 units of unfractionated heparin, 1 mg verapamil, and 200 mcg nitroglycerin were injected through the sheath. Midazolam 1 mg intravenously was administered at the operator's discretion in anxious patients. Puncture time was noted from the time local anaesthetic was infiltrated till successful insertion of radial sheath.

CAG and PCI were performed by the usual techniques and further doses of heparin were administered during PCI to maintain target activated clotting time (ACT). Procedure duration, fluoroscopic time, and radiation dose area product (DAP) were noted.

**Figure 3:**
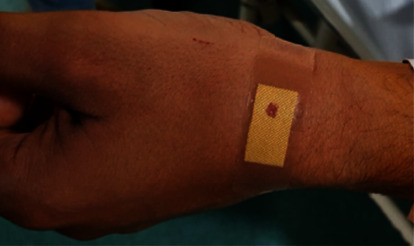
Posthaemostasis noncompressive tape over puncture site.

After the procedure, radial sheath was withdrawn 2–3 cm from the puncture site. A 5 × 5 cm gauze pad was placed over the puncture site, following which the sheath was removed completely. Manual compression was then applied over gauze pad by hand till complete haemostasis was achieved. Subsequently, the puncture site was covered with a noncompressive tape (Figure 3). Compression was provided by experienced cath lab personnel who learnt about the procedure beforehand. Optimum pressure and free space were ensured to permit venous outflow. Local examination was performed every hour for three hours to look for arterial pulse and development of any new local complication(s). In the event of puncture site haematoma, further manual compression was applied till complete haemostasis was achieved.

After discharge, patients were reevaluated at one and four weeks. Whenever possible, LRA patency was assessed using reverse Barbeau test. This is performed by compressing ipsilateral ulnar artery at the wrist with concomitant oximetric evaluation of radial arterial flow waveform [7].

### 2.3. Study End Points

The primary study endpoints were as follows:

- Coronary angiography success (%), defined as optimal visualization of both coronary arteries and branches
- Percutaneous coronary intervention success (%), defined as achievement of target vessel patency with <20% residual stenosis

The secondary study endpoints were as follows:

- Puncture success of left distal radial artery (%)
- Diameter of left distal radial artery, measured by preprocedure ultrasonography (mm)
- Postprocedure puncture site haemostasis duration (minutes), defined as time from radial sheath removal till complete cessation of puncture site blood flow
- Frequency rate of puncture site complications (%)
- Procedure time, including coronary angiography and percutaneous coronary intervention, if performed (minutes)
- Total radiation exposure time during procedure (minutes)
- Dose of radiation (dose area product: DAP) during procedure (Gray-cm^2^)
- Degree of patient satisfaction using a visual analogue scale for pain and an overall satisfaction score (in a scale of 1–10, ≥7 for severe pain, and ≤3 for unsatisfactory procedure)

Bleeding complications were classified according to Bleeding Academic Research Consortium (BARC) classification [8]. Puncture site haematoma was categorized as <2 cm, 2–5 cm, or >5 cm extension from the puncture site.

A comparative evaluation of the procedural characteristics of ldTRA and RRA access routes was undertaken. Study site patients who underwent RRA route procedures on the same day in the particular cath lab as the ldTRA procedures were included in the analysis. Procedure time, fluoroscopic time, and radiation DAP were compared between the two groups.

### 2.4. Statistical Analysis

Continuous variables were expressed as mean ± standard deviation or median with interquartile range (IQR). Categorical variables were expressed as percentages. Normality of data was assessed using Shapiro-Wilk and Kolmogorov-Smirnov tests. Quantitative comparative study was done using Student's *t*-test or Mann-Whitney *U* test, as indicated by normality of data distribution. A two-sided *p* value of 0.05 was of statistical significance. All statistical analyses were performed using IBM SPSS Statistics for Windows, version 28.0.0.0 (IBM Corp., Armonk, N.Y., USA).

## 3. Results

**Figure 4:**
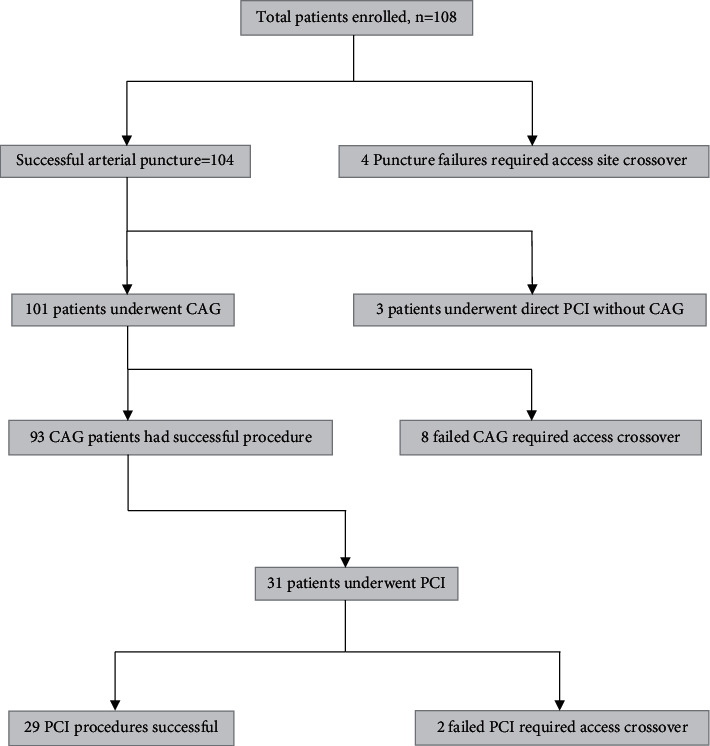
Flowchart depicting patient distribution in the study.

Between April 2018 and June 2020, 108 consecutive patients were enrolled for the study (Figure 4). The target number of patients for enrolment was 200, but due to slow recruitment, the study had to be terminated early, with 108 patients being able to complete the study. The preprocedural baseline characteristics are tabulated in Table 1. Mean patient age was 57.7 ± 11.4 years with 68.5% males and 31.5% females. Mean body mass index was 28.9 ± 5.9 kg/m^2^. 70.4% patients were hypertensive, 54.6% had diabetes mellitus, and 24.1% had current smoking history. History of dyslipidaemia was found in 49.1% of patients, while 1.9% patients had history of chronic kidney disease (CKD). Acute coronary syndrome (ACS) as presentation was noted in 53.7% patients [0.9% ST elevation myocardial infarction (STEMI) and 52.8% non-ST elevation ACS].

**Figure 5:**
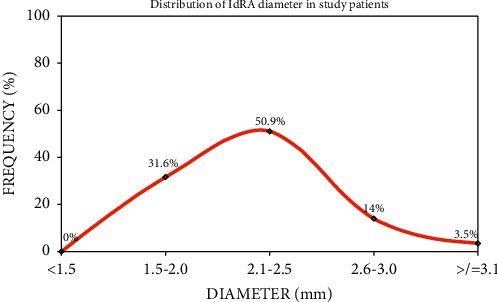
Left distal radial artery diameter among patients.

Mean ldRA diameter was 2.25 ± 0.34 mm with a median of 2.2 mm [IQR-0.2] (Figure 5). Successful arterial puncture was performed in 104/108 patients (96.3%). Mean puncture time was 6.7 ± 7.1 minutes. In the primary outcome measures (Table 2), CAG was attempted in 101 patients and was successful in 93 patients (92.1%), while PCI was successful in 32 of 34 patients (94.1%). Crossover rate to alternate access site was 14/108 (13.0%). Coronary artery disease (CAD) extent in CAG patients were triple vessel disease, 14/93 (15.1%), double vessel disease, 17/93 (18.3%), single vessel disease, 21/93 (22.6%), left main disease, 4/93 (4.3%), and noncritical disease, 19/93 (20.4%). Fractional flow reserve (FFR) was assessed in 3/32 procedures (9.4%).

Among secondary outcome measures, mean procedure time was 55.7 ± 32.8 minutes. Median total fluoroscopic time was 6.6 minutes [IQR-14.2] and a median total radiation DAP of 39.2 Gy-cm^2^ [IQR-97.0]. Mean haemostasis duration was 23.1 ± 11.9 minutes.

Iohexol was the contrast agent used in all patients undergoing CAG/PCI, except in one patient of CKD in whom iodixanol was used. Mean contrast volume used was 112.5 ± 97.6 ml, with 53.8 ± 32.3 ml during CAG and 191.8 ± 74.3 ml during PCI. 5F diagnostic catheters were used in 92 CAG procedures (98.9%), while 6F catheter used in a single procedure (1.1%). Two different types of catheters were used in 2/93 procedures (2.2%), while a single type of catheter was used in the rest (91/93, 97.8%). During PCI, 6F guide catheters were the most frequently used (24/32, 75.0%) followed by 5F catheters (10/32, 31.3%). 7F guide catheter was used in a single procedure (1/32, 3.1%).

There were four puncture failures, eight failed CAG, and two failed PCI procedures, which required crossover to alternative access site (Table 3). Most failed procedures were due to radial arterial spasm (8/14, 57.1%). In one patient, there was embolization of guidewire into LRA that was retrieved successfully. Another patient underwent successful left coronary cannulation via ldTRA access, but right coronary cannulation failed due to arterial spasm.

Among the patients in the right radial group (*n* = 121), mean age was 59.3 ± 10.4 years with 71.1% males and 28.9% females (Table 1). 60.3% of patients had a diagnosis of non-ST elevation acute coronary syndrome. One hundred twenty-one patients underwent CAG, while 33 patients underwent PCI. FFR study was performed in one patient. There was no alternative access site requirement in any of the procedures. Coronary artery disease distribution observed was triple vessel disease, 22/121 (18.2%), double vessel disease, 23/121 (19.0%), single vessel disease, 28/121 (23.1%), left main disease, 2/121 (1.7%), and noncritical disease, 24/121 (19.8%).

**Figure 6:**
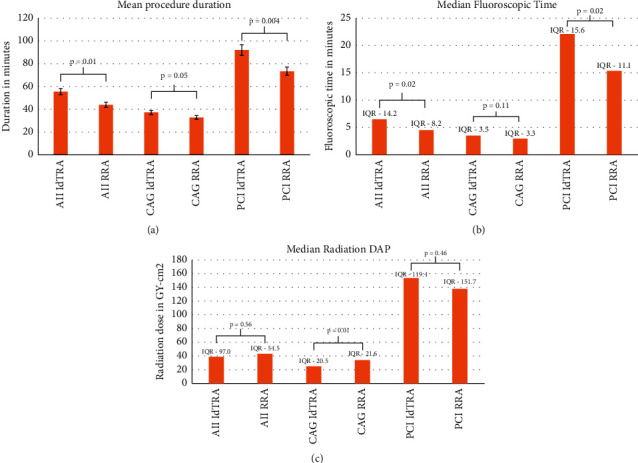
(a) Comparison of left distal radial and right radial mean procedure duration, (b) median fluoroscopic time during left distal radial and right radial procedures, and (c) median radiation dose (DAP) during left distal and right radial procedures.

Comparative analysis (Table 4) of ldTRA access with RRA access revealed significantly higher mean procedure time, 55.7 ± 32.8 vs. 43.9 ± 26.0 minutes (*p* = 0.01), and median total fluoroscopic time, 6.6 minutes [IQR: 14.2] vs. 4.7 minutes [IQR: 8.2] (*p* = 0.02) in the former (Figure 6(a) and 6(b)), driven by a significant difference during PCI. There was no significant difference in median total radiation DAP between the two access sites (Figure 6(c)), 39.2 Gy-cm^2^ [IQR-97.0] vs. 43.8 Gy-cm^2^ [IQR-54.5] (*p*=0.56). Median radiation DAP during CAG was significantly lesser in ldTRA cohort compared to RRA cohort (Figure 6(c)), 26.2 Gy-cm^2^ [IQR-20.5] vs. 34.9 Gy-cm^2^ [IQR-21.6] (*p*=0.01).

**Figure 7:**
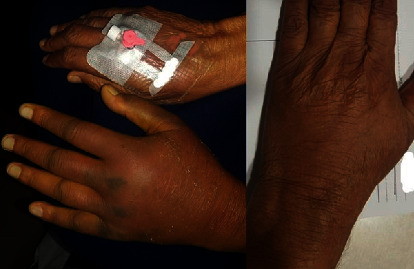
Postprocedure major haematoma in a patient, which resolved after three-week duration.

Puncture site complications (Table 5) were noted in 11 patients (10.2%). None had BARC type 2, 3, or 5 bleeding. Two patients (1.9%) had >5 cm haematoma, which resolved completely by three weeks (Figure 7). Three patients (2.8%) had 2–5 cm haematoma, while six patients (5.5%) had <2 cm haematoma. All nine patients had complete healing during hospital stay. There was no incidence of radial artery loss, dissection, pseudoaneurysm, arteriovenous fistula formation, or nerve injury.

Postprocedure visual analogue scoring for pain and satisfaction was assessed in English or Hindi language. Mean pain and satisfaction scores were 2.4 ± 2.3 and 9.0 ± 1.3, respectively. 7.7% patients reported a pain score of ≥7, while 3.8% reported a satisfaction score of <4. There were no deaths reported during the study period.

## 4. Discussion

Femoral artery has traditionally been the default route for coronary intervention; however, TRA has increased in recent times due to improved clinical outcomes and patient comfort compared with transfemoral approach [9]. Most operators prefer RRA for transradial procedures because they are usually right-handed and comfortable using this route. Our institution has been performing transradial procedures since 2006 (1000 procedures/year).

Conversely, the LRA approach offers several advantages. Due to the anatomical position, catheter manipulation is like femoral approach. Left brachial and subclavian arteries are less tortuous compared with right side and provide more direct access to aortic root [10]. Since most people are right-handed, postprocedure compression on left hand is more comfortable for the patient. Comparative studies on RRA and LRA access have shown similar efficacy and safety in clinical outcomes [10]. LRA access has been shown to be associated with lower incidence of in-hospital stroke, lower fluoroscopic time, and contrast use. [11, 12]

LdTRA is a novel access site that has recently been used with acceptable safety and efficacy [13, 14]. Distal radial artery in the anatomical snuff box is quite superficial and this area is also devoid of vital veins or nerves. Hence, vascular access in this location may have reduced risk of bleeding or nerve injury while also sparing the proximal arterial segment.

However, some unresolved issues remain. The learning curve needs to be mastered since puncture may not be possible when the artery is not palpable. Also, there is no universally accepted method for postprocedure haemostasis. Limited studies dwell on these questions in medical literature [4, 5]. Previous case studies of ldTRA access have reported results involving sample size of up to 200 patients [4]. Our study was also designed along these lines to assess the results among Indian patients. The purpose of this open level, prospective, observational cohort study was to assess the safety and feasibility of ldTRA for CAG and PCI.

Majority of our patients had ACS with increased prevalence of CAD risk factors. Success rates of arterial puncture, CAG, and PCI are comparable or better than previous studies [13, 15]. This can be ascribed to our rigorous protocol of ultrasound guided vascular puncture. Excessive manipulation and pricks to the artery predisposes to arterial spasm, while a through-and-through puncture is painful and causes haematoma formation [16]. Using our method, only two procedures required an anatomical snuff box puncture, while rest all were performed in the distal arterial segment. Since 5 F arterial sheath (2.29 mm outer diameter) was initially used in most patients (mean ldRA diameter 2.25 ± 0.34 mm), this could have contributed to puncture success. Increased sheath/artery diameter ratio (SAR) has been associated with procedure failure [17].

Haemostasis was accomplished by manual compression in our study. We selected this method due to various reasons. Suitable anatomy helps in adequate compression being applied, while the hand can be monitored easily for vascular leak or swelling. Being the nondominant hand in majority, patient cooperation and comfort is achieved. However, manual compression requires meticulous attention as the artery may slip over underlying bony prominence, resulting in vascular leak. It is also labour intensive, requiring experienced, dedicated cath lab personnel. Despite these challenges, our study revealed a mean haemostasis duration of 23.1 ± 11.9 minutes with 10.2% incidence of local haematoma, which resolved completely in all patients on follow-up. Deep seated artery, multiple punctures, friable skin, use of dual antiplatelet agents, and inappropriate compression could be some of the reasons associated with local vascular complications. Various studies have reported different methods of haemostasis with ldTRA, from manual compression to dedicated compression devices [18, 19]. However, current evidence indicates relatively uncomplicated haemostasis with ldTRA, usually within three hours of procedure completion [4]. A clinically validated haemostasis protocol and specific compression devices are required for wider application of this technique.

Comparative study with RRA procedures revealed, higher procedure and fluoroscopic time with ldTRA, driven by PCI procedures (Figure 6). These findings could be explained by the fact that with any new access site, proficiency is gained with experience, as was seen earlier when TRA was first introduced [20]. Reduced puncture time and procedure duration have been reported after gaining experience with ldTRA [13]. More complex CAD subset in ldTRA cohort could also have led to these findings in our study.

Similar radiation dose between both access sites, with a reduced dose in ldTRA CAG cohort, provides evidence of patient safety with this technique. In a meta-analysis involving 22 studies with 10,287 patients, LRA access was associated with significantly lower fluoroscopic time with no significant difference in radiation DAP [10]. In another study involving 1,467 patients, LRA access was associated with significantly lower fluoroscopic time and radiation DAP compared to RRA access during CAG, with no significant difference during PCI [21]. Our findings are in line with these previous studies.

Most procedure failures in our study were due to radial artery spasm (57.1%), mostly during instrumentation. Radial artery spasm is one of the commonest complications of transradial procedures, with a 4-20% incidence and contributing to procedure failure in 38% cases [17, 22]. Factors contributing to radial spasm are enumerated in Table 6. Increased periprocedural anxiety associated with a novel access site, multiple puncture attempts and a distinct learning curve for gaining proficiency in ldRA puncture could be some of the reasons for the incidence of radial arterial spasm in our patient cohort. Detailed preprocedure patient counselling, imaging guided puncture, and use of effective spasmolytic medications could have led to access site abandonment in only 5 of the 101 patients undergoing CAG and 2 of 31 patients undergoing PCI. As proficiency is gained in the ldTRA puncture technique, further studies focusing on incidence of radial arterial spasm would help to ascertain the rates of procedure failure due to this complication. There were no other arterial complications noted in our patients during one month of follow-up.

Less periprocedural pain and overall high patient satisfaction were noted throughout this study. Avoidance of multiple pricks and through-and-through puncture is helpful for ensuring patient comfort. Inadvertent needle injury of underlying periosteum causes severe pain and predisposes to arterial spasm [16]. Imaging guidance and maintaining a 30-degree puncture needle angle helps in preventing this complication. During ldTRA procedure, a pronated, flexed left arm resting on the abdomen is a more comfortable posture for the patient. After the procedure, the patient is free to use his/her right hand while compression is provided on left hand. All these factors may explain high procedure satisfaction in this patient subset.

### 4.1. Study Limitations

This was an observational study at a single centre that requires confirmation from multicentre studies. Significant difference between ldTRA and RRA access can best be assessed with a randomized control trial. We had only a single STEMI patient undergoing primary PCI via ldTRA access, though there was no significant effect on the door to balloon time. However, further studies are needed to evaluate feasibility and door to balloon times during ldTRA PCI procedures. Finally, we could not assess our patients by ultrasound and doppler evaluation for radial artery flow during follow-up. Though thorough clinical evaluation and reverse Barbeau test was performed during follow-up, an ultrasonographic evaluation could have better diagnosed radial artery patency in these patients.

## 5. Conclusions

The present study has demonstrated feasibility and safety of ldTRA as a route for coronary interventions. Assessment of radial artery palpability and local anatomy is the key to ensure puncture success. Ultrasound guided vascular puncture was accompanied by high success and low rate of complications. Radial artery spasm was the most frequent complication noted. A distal radial artery diameter of ≥2 mm was associated with favourable outcomes in our study. Greater patient comfort and satisfaction was noted with this access route. Multicentric and comparative studies with RRA access will establish ldTRA route as a viable option in coronary interventions.

## 6. Impact on Daily Practice

LdTRA coronary intervention technique is feasible with acceptable safety margin. The technique has a learning curve, accomplishing which increases procedural success. Ultrasound guidance ensures successful vascular puncture and lower complications. Manual haemostasis is effective in ldTRA due to anatomic factors and patient cooperation. With experienced operators, fluoroscopic time and radiation dose are similar to RRA access.

## Figures and Tables

**Table 1:** Baseline characteristics of patients.

	Left distal radial access	Right radial access	*p* value
Study patients (n)	108	121	---
Mean age (years)	57.7 ± 11.4	59.3 ± 10.4	0.27
Males (%)	68.5	71.1	0.77
Previous risk factors			
Hypertension, *n* (%)	76 (70.4)	82 (67.8)	0.78
Diabetes mellitus, *n* (%)	59 (54.6)	69 (57.0)	0.79
Current smoking, *n* (%)	26 (24.1)	36 (29.8)	0.37
Dyslipidaemia, *n* (%)	53 (49.1)	62 (51.2)	0.79
Chronic kidney disease, *n* (%)	2 (1.9)	4 (3.3)	0.69
Prior PCI, *n* (%)	7 (6.5)	8 (6.6)	1.00
Prior CABG, *n* (%)	2 (1.9)	3 (2.5)	1.00
Provisional diagnosis			
NSTE-ACS, *n* (%)	57 (52.8)	73 (60.3)	0.29
Chronic stable angina, *n* (%)	8 (7.4)	12 (9.9)	0.64
STEMI, *n* (%)	1 (0.9)	22 (18.2)	0.0001
Others, *n* (%)	42 (38.9)	14 (11.6)	0.0001

PCI: percutaneous coronary intervention; CABG: coronary artery bypass graft; NSTE-ACS: non-ST elevation acute coronary syndrome; STEMI: ST elevation myocardial infarction.

**Table 2:** Outcomes of study.

Primary outcomes	
Coronary angiography success, *n* (%)	93/101 (92.1)
Percutaneous coronary intervention success, *n* (%)	32/34 (94.1)
Secondary outcomes	
Puncture success of left distal radial artery, *n* (%)	104/108 (96.3)
Diameter of left distal radial artery by ultrasound (mm)	2.25 ± 0.34 (mean) 2.20 (median), IQR-0.2
Mean haemostasis duration (minutes)	23.1 ± 11.9
Puncture site complication, *n* (%)	11/108 (10.2)
Mean procedure time (minutes)	55.7 ± 32.8
Median total fluoroscopic time (minutes)	6.6 (IQR-14.2)
Median total radiation dose area product (Gray-cm^2^)	39.2 (IQR-97.0)
Mean visual analogue scale for pain score (0–10)	2.4 ± 2.3
Pain score ≥7 (%)	7.7
Mean patient satisfaction score (0–10)	9.0 ± 1.3
Satisfaction score <4(%)	3.8

mm: millimetre; cm: centimetre.

**Table 3:** Procedure failures during the study.

Vascular puncture			
Patient	Cause	ldRA diameter	Alternative access
1	Small artery (needle instability)	1.9	RRA
2	Small collapsed artery	2.0	RRA
3	Small artery with spasm	1.8	RFA
4	Deep seated artery (needle instability)	2.0	LRA
Coronary angiography			
	Sheath/ldRA ratio		
1	Bilateral radial artery tortuosity	0.76	RFA
2	Failed catheter access (spasm)	1.0	RRA
3	Failed catheter access (spasm)	1.2	LRA
4	No arterial sheath backflow due to spasm	0.9	RRA
5	Guidewire embolization	0.7	LRA
6	Failed catheter access (spasm)	1.3	RRA
7	Left radial artery spasm (partial failure)	1.1	LFA
8	Failed catheter access (peripheral arterial disease)	1.1	RRA
Percutaneous coronary intervention			
		Sheath/ldRA ratio	
1	Left radial artery spasm	1.0	RRA
2	Left radial artery spasm	1.1	RRA

ldRA: left distal radial artery; LFA: left femoral artery; RFA: right femoral artery; RRA: right radial artery; LRA: left radial artery.

**Table 4:** Comparison between left distal radial and right radial access procedures.

	Left distal radial access	Right radial access	*p* value
Mean procedure time in minutes with standard deviation			
Total	55.7 ± 32.8	43.9 ± 26.0	0.01
CAG	37.3 ± 15.4	32.9 ± 16.1	0.05
PCI	92.1 ± 27.3	73.4 ± 24.7	0.004
Median fluoroscopic time in minutes with interquartile range			
Total	6.6 (14.2)	4.7 (8.2)	0.02
CAG	3.6 (3.5)	3.1 (3.3)	0.11
PCI	22.3 (15.6)	15.4 (11.1)	0.02
Median radiation dose area product in Gray-cm^2^ with interquartile range			
Total	39.2 (97.0)	43.8 (54.5)	0.56
CAG	26.2 (20.5)	34.9 (21.6)	0.01
PCI	153.3 (119.4)	137.7 (151.7)	0.46

CAG: coronary angiography; PCI: percutaneous coronary intervention; cm: centimetre.

**Table 5:** Puncture site complications.

BARC bleeding types 2 ,3, 5	0
Local haematoma, *n* (%)	11/108 (10.2)
Haematoma grade	
<2 cm, *n* (%)	6/108 (5.5)
2–5 cm, *n* (%)	3/108 (2.8)
>5 cm, *n* (%)	2/108 (1.9)
Arterial complications	
Distal radial arterial loss	0
Dissection	0
Pseudoaneurysm	0
Perforation	0
Arteriovenous fistula	0
Guidewire embolization	1
Follow-up	
Distal radial arterial loss	0
Nerve injury	0

BARC: Bleeding Academic Research Consortium.

**Table 6:** Factors contributing to Radial spasm.

Small sized artery
Anatomic anomalies (radial loop, high take-off)
SAR>1
Female gender
Multiple catheter use
Repeated punctures
Moderate to severe procedural pain
Patient anxiety

SAR: sheath outer diameter/artery ratio.

## Data Availability

The datasets used during the current study are available from the corresponding author upon reasonable request.

## Abbreviations

TRA: Transradial approach
STEMI: ST elevation myocardial infarction
ldTRA: Left distal transradial artery approach
CAD: Coronary artery disease
LRA: Left radial artery
RRA: Right radial artery
SAR: Sheath/artery diameter ratio
CABG: Coronary artery bypass graft
ldRA: Left distal radial artery
VKLITE: Vasant Kunj Left dIstal Transradial artEry approach
CAG: Coronary angiography
PCI: Percutaneous coronary intervention
cc: Cubic centimetre
F: French
mg: Milligram
mcg: Microgram
ACT: Activated clotting time
DAP: Dose area product
mm: Millimetre
BARC: Bleeding Academic Research Consortium
cm: Centimetre
IQR: Interquartile range
CKD: Chronic kidney disease.
